# Misleading health risk information and public health decision-making on digital platforms: evidence from tech-fearmongering food production short videos

**DOI:** 10.3389/fpubh.2026.1894652

**Published:** 2026-07-20

**Authors:** Qiong Zhao, Haochen Yu, Xiaoyu Chen

**Affiliations:** 1 Shanghai Institute of Visual Arts, Shanghai, China; 2 Social Research Institute, University College London, London, United Kingdom; 3 Law School, Peking University, Beijing, China

**Keywords:** digital public health, health risk information, negative emotions, platform governance, protective behavioral intention, public health decision-making, risk communication, trust erosion

## Abstract

**Background:**

Digital platforms have become important channels for public health risk information and everyday health decision-making. Misleading risk information may distort public risk cognition, weaken trust in regulatory systems, and reshape protective behavioral intentions.

**Methods:**

Grounded in Protection Motivation Theory, this study examined tech-fearmongering food production short videos as a case of misleading health risk information in platform environments. A scenario-based experimental design was used with 487 valid responses from Chinese internet users. Single-mediator and parallel multiple mediator models were estimated using PROCESS Model 4 with 5,000 bootstrap resamples to test the mediating roles of health threat appraisal, negative emotions, and trust erosion.

**Results:**

Cognitive, affective, and social trust pathways all contributed to the mechanism linking misleading risk information to public health decision-making. In the parallel mediation model, health threat appraisal and trust erosion showed unique significant indirect effects on public risk cognition, while health threat appraisal was the dominant unique mediator for protective behavioral intention.

**Conclusion:**

Tech-fearmongering food production short videos can influence public health decision-making through health threat appraisal, negative emotions, and trust erosion. Digital health risk information governance should address emotional reactions and trust erosion in addition to cognitive threat appraisal, with implications for platform governance, health risk communication, and public health information quality control.

## 1 Introduction

The proliferation of digital platforms has reshaped how the public accesses and interprets health risk information. Short-video platforms such as Douyin and Kuaishou have become important channels through which users encounter food-related health information. From a digital public health perspective, this creates both communication opportunities and governance risks. Some user-generated content exploits public anxiety about food safety (1) and limited food science knowledge (2) to circulate misleading risk information (3), which may affect public health judgments and protective behavioral intentions (4, 5). Tech-fearmongering food production short videos are a visible example of this problem. These videos present non-food industrial imagery, misuse chemical terminology, construct an industrial threat narrative, and reduce food science to a binary opposition between natural and technological. In doing so, they may undermine trust in food safety governance and distort public health decision-making. This constitutes a public health information governance challenge in the digital era.

Protection Motivation Theory (PMT) provides a classic framework for understanding how such fear appeals trigger protective intentions (6, 7). The theory asserts that cognitive processes, rather than fear itself, are the mediators of behavior change (8). However, this cognitive-centric interpretive model shows its limitations when explaining tech-fearmongering food production short videos, which are highly dependent on visual impact and emotional resonance: on the one hand, strong emotions may play an independent role beyond cognitive assessment (9); on the other hand, the systematic trust eroded by video content disintegrates the social foundation for effective response evaluation in PMT.

Therefore, this study does not simply apply PMT to a new communication case. It uses misleading food-related health risk information as an empirical setting to test how cognitive appraisal, negative emotion, and trust erosion jointly shape public health decision-making on digital platforms. The findings are expected to inform digital public health risk governance, platform content review, and risk communication strategies.

## 2 Literature review

To build the theoretical basis of this study and clearly define its theoretical contribution, this paper reviews the existing literature and finds that most research focuses on single mechanisms of misinformation impact, lacking investigation into the multi-pathway mechanisms specific to tech-fearmongering food production short videos.

Social media platforms, particularly short-video services, have become major vectors for the dissemination of food safety misinformation. Due to the lack of gatekeepers and fact-verification mechanisms, the internet has further facilitated the spread of false information (10), and social media has gradually become a hotbed for misinformation (11–13). Some content creators skillfully employ conspiracy theory narratives and logic (14–17) to produce misleading food safety videos and disseminate them on short-video platforms. These videos often fabricate rich narratives, use anecdotes or personal experience, wrap content in the guise of entertainment or secret-revealing, improperly use scientific evidence, exploit public emotions, and pass biased reasoning to the audience (18, 19), inciting distrust of the government and the food industry system (20). The essence of such content is inflammatory food safety panic marketing disguised as entertainment. Influenced by cognitive inertia (21), consumers do not always critically analyze received information (22) and may even internalize misleading information markers (23).

### 2.1 The impact of food safety misinformation on cognitive risk appraisal

Social media amplifies the public’s food safety risk perception (FSRP). As an individual’s subjective assessment of potential food hazards, FSRP is influenced by knowledge level, media exposure, and socio-demographic characteristics (24). Social media magnifies perceived food safety risks through increased attention and extensive coverage (25, 26). Research demonstrates that media exposure to food scandals or unconventional production processes significantly increases public risk perception and safety concerns (1). Due to insufficient food science knowledge (27), the public tends to have heightened risk perceptions of food additives and new food technologies, often attributing food safety consequences of illegal acts to food additives or new technologies themselves (2).

Tech-fearmongering food production short videos affect public judgment by activating the threat appraisal mechanism. By deliberately emphasizing artificial synthesis, industrial manufacturing, and chemical addition, these videos encourage the public to interpret misleading content as disclosure of food scandals and to equate such descriptions with health threats. Such content exploits public vigilance toward unknown technologies and additives (28), stimulates threat appraisal, affects macro safety judgments of daily food, increases individual health threat appraisal regarding food additives and the broader food supply chain, and encourages protective actions (7).

### 2.2 The impact of food safety misinformation on affective responses

The impact of food safety misinformation extends far beyond the cognitive level, frequently employing emotional content to enhance communication and persuasion. Emotional appeal is a common persuasion strategy in misinformation (9), and exposure to false information directly elicits negative emotions (29), which can significantly mediate the impact of misinformation on cognition and behavior (3, 30). Emotional information can directly dominate individual judgment and evaluation (31). Under the influence of negative emotions, the public develops more vivid but less accurate memories of related events (32), becoming more prone to cognitive errors.

In decision-making processes, emotional states often exert greater influence than logical factors, with research suggesting that 95% of consumption decisions are essentially driven by emotional factors [as cited in (4)]. Fear, as a typical negative emotion (33), shows particularly strong influence in health risk contexts (34, 35). But in extreme cases, fear can directly affect behavioral decisions even beyond the cognitive assessment process (36). According to the Affect Heuristic Theory, in complex and uncertain decision-making situations such as those presented by tech-fearmongering food production short videos, individual emotional experience plays a stronger guiding role than pure rational analysis (37). This study posits that negative emotions serve not only as a key variable in information dissemination (30), but also as a mediating pathway independent of cognitive assessment (3)—a key revision of PMT’s cognitive-centric framework.

### 2.3 The impact of food safety misinformation on social trust

Trust is the psychological foundation for the public’s acceptance of food safety information and technology. Consumer trust in food safety encompasses brand trust and system trust (38–40). System trust—trust in government and institutions—is positively correlated with consumer confidence in food safety (38, 41) and serves as a key factor in reducing public risk perception and improving food acceptance (2). Food safety misinformation systematically erodes the public’s trust system through conspiracy theory narratives and logic (14, 42, 43). According to PMT, the collapse of trust directly undermines the individual’s response evaluation process. Once trust is damaged, individuals are more likely to adopt skeptical stances, amplify risk perception (44), and engage in avoidance behavior. Research in China further demonstrates that lack of organizational trust aggravates public concern about food safety (45). Therefore, this study conceptualizes trust erosion as a key social barrier to response evaluationin the PMT framework.

Trust erosion affects not only cognition but also behavioral intentions (46). Research shows that trust in media-provided food safety information directly affects purchase intention (47), and in the context of natural food, lack of brand trust and system trust directly influences protective behavior intention (20).

## 3 Research design

According to PMT, when individuals perceive a threat, they undergo two core cognitive processes: threat assessment and response evaluation (6), generating protective motivation that drives protective behavior (48, 49). However, classical PMT lacks explanatory power in three key areas—risk levels, emotional mechanisms, and social foundations—when explaining complex social food safety risk information such as tech-fearmongering food production short videos. To address these limitations, this study builds upon the core logic of PMT to construct an integrative theoretical model.

### 3.1 Research hypotheses

To examine how tech-fearmongering food production short videos influence consumers’ risk perception and protective behavioral intentions, it is first necessary to clarify the core concepts. The independent variable in this study is tech-fearmongering food production short videos, which refers to food-related short videos that use non-food industrial imagery, vague chemical terminology, and high-tech or heavy-duty production cues to construct a misleading health risk narrative. As external threat information, this variable serves as the initial stimulus that triggers individuals’ threat appraisal and response evaluation.

Since it is difficult to directly measure actual protective behaviors in cross-sectional surveys, and behavioral intentions have been widely confirmed as direct antecedents and effective proxy indicators of actual behaviors, this study will focus on exploring the mechanisms through which misleading short videos influence intentions to engage in protective behaviors. The dependent variables in this study are public risk cognition, the relatively stable, comprehensive, and macro-level judgment individuals form regarding the overall food safety situation after watching tech-fearmongering food production short videos, which serves as a crucial foundation for public health decision-making, and protective behavior intention, which refers to an individual’s propensity to take specific actions to avoid perceived food safety risks.

#### 3.1.1 Health threat appraisal as the primary cognitive pathway linking tech-fearmongering food production short videos to public reactions

The study operationalizes the core cognitive process of threat appraisal in PMT as individual health threat appraisal, referring to the degree of subjective evaluation and concern individuals have regarding the potential harm to their own and their family’s health after reading the tech-fearmongering food production scenario. Distinguished from the macro perception of public risk cognition, this variable focuses on judgments of the severity and susceptibility of specific health threats inspired by food-related content, representing the micro and immediate level of threat appraisal.

This operation has a solid theoretical and empirical basis. Short videos play an important role in risk communication (25, 26, 50). When tech-fearmongering food production short videos deliberately magnifies the potential hazards of food additives through visual impact and terminology barriers, it deliberately transmits threat information about food sources and safety, and magnifies the threat assessment of the public (2). According to the Perceived Threat Model (PMT), an activated threat assessment is the most direct driver of protective motivation [as cited in (60)]. By exaggerating the harm (severity) of food additives and implying their ubiquity (vulnerability), short videos directly increase individuals’ health threat assessments (2). According to the PMT, an elevated threat assessment subsequently positively influences the public’s macro-level risk perception (1, 2, 44, 51) and drives them to adopt protective behaviors (7, 52–55). Therefore, this study proposes:

*H1*: tech-fearmongering food production short videos have a positive effect on individual health threat appraisal.

*H1a*: tech-fearmongering food production short videos indirectly and positively influence public risk cognition through individual health threat appraisal.

*H1b*: tech-fearmongering food production short videos indirectly and positively influence protective behavior intention through individual health threat appraisal.

#### 3.1.2 Negative emotions serve as the emotional pathway linking tech-fearmongering food production short videos to public reactions

This study established negative emotions as the key emotional path in the theoretical framework of protective motivation, which is an important expansion of the classical theory. Negative emotions refer to the emotional reactions such as anxiety and fear caused by watching short videos, which are independent of cognitive assessment.

The setting of this path is supported by the Affect Heuristic Theory of risk decision research. The theory emphasizes that in complex and uncertain decision-making situations, individual emotional experience plays a stronger leading role in individual behavior than pure rational analysis (37). This view echoes the “risk is emotion” hypothesis proposed by Loewenstein et al. In the early stage, which also emphasizes the core position that expected emotions (such as fear and anxiety) can go beyond cognitive assessment and directly drive behavioral decision-making (56). In the field of food safety, studies have confirmed that fear can significantly affect consumers’ food choices and directly promote them to take protective consumption behavior (4). On this basis, this study believes that tech-fearmongering food production short videos deliberately uses the negative emotions of the public through emotional narration similar to conspiracy theory (4), thereby affecting individual risk cognition and behavior intention (3). Therefore, this study proposes that:

*H2*: tech-fearmongering food production short videos have a positive effect on negative emotions.

*H2a*: tech-fearmongering food production short videos indirectly and positively influence public risk cognition through negative emotions.

*H2b*: tech-fearmongering food production short videos indirectly and positively influence protective behavior intention through negative emotions.

#### 3.1.3 Trust erosion as the social mediating pathway linking tech-fearmongering food production short videos to public reactions

This study conceptualizes trust erosion as a socialized extension of the coping evaluation component within PMT. This variable refers to the extent to which individuals’ trust in food regulatory systems, food producers, and information channels diminishes after watching short videos.

This pathway aims to overcome the explanatory limitations of classical theories at the societal system level. When tech-fearmongering food production short videos erodes public system trust and brand trust through implication, system trust not only directly influences individuals’ judgment of misinformation but also positively moderates the protective role of brand trust; when systemic trust is absent, the protective role of brand trust is significantly weakened, leading individuals to harbor dual doubts regarding the efficacy of official measures and their own self-protection capabilities (20). In other words, tech-fearmongering food production short video content simultaneously undermines both the response efficacy (44) and self-efficacy (57) posited in the Protection Motivation Theory.

Relevant research provides strong support for this pathway. Short videos depicting “food produced by technology” severely undermine public trust in the regulatory system and food manufacturers by spreading information that fosters distrust (20, 44). According to the PMT, confidence in the efficacy of recommended response measures is key to adopting protective behaviors. A lack of trust directly undermines this belief in efficacy, causing individuals to amplify their perception of risk, even when they perceive a threat, because they do not believe the system or themselves can respond effectively, and thus making them more inclined to adopt self-protective behaviors. Therefore, this study proposes:

*H3*: tech-fearmongering food production short videos have a positive effect on trust erosion.

*H3a*: tech-fearmongering food production short videos indirectly and positively influence public risk cognition through trust erosion.

*H3b*: tech-fearmongering food production short videos indirectly and positively influence protective behavior intention through trust erosion.

### 3.2 Model building

**Figure 1:**
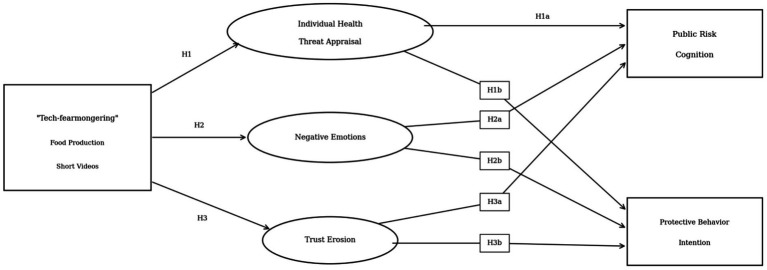
Schematic diagram illustrating the mechanism by which tech-fearmongering food production short videos influence the public's risk perception and intention to adopt protective behaviors.

Based on the aforementioned theories, the independent variable tech-fearmongering food production short videos influences the dependent variables public risk cognition and protective behavior intention through three parallel mediating variables: individual health threat appraisal, negative emotions, and trust erosion (Figure 1).

### 3.3 Scale design and questionnaire sample selection

To examine the short-term effects of tech-fearmongering food production short videos under controlled exposure conditions, this study employed a scenario-based experimental design and quantitative analysis. Two food preparation video descriptions were selected from the Douyin platform and adapted as experimental and control scenarios:

*Experimental group*: participants read a description of a food preparation video characterized by tech-fearmongering. In the video, the content creator stands in front of a food cart with dozens of test tubes and glass jars, each labeled with a chemical name. The blogger uses numerous food additive names and chemical terms during the preparation process.

*Control group*: participants read a description of a normal food preparation video. The blogger operates in a shop kitchen with common kitchen utensils and natural food materials. The production process emphasizes heat, time, and natural flavor, clearly showing all changes from raw materials to finished products.

The core scales were adapted from empirically validated instruments and contextualized for the study of misleading food-related health risk information on short-video platforms. Two experts reviewed content validity, and the research team conducted several rounds of revision to improve clarity and logical coherence. All core items were scored on a five-point Likert scale, from 1, strongly disagree, to 5, strongly agree. The study measured five core constructs with the following operational definitions and scale sources:

*Negative emotions (NE)*: negative emotional states such as anxiety, disgust, and a sense of being deceived experienced by individuals after watching the video. This scale is adapted from the emotional dimension of the DASS-21 scale (58) and consists of three items after item purification (Table 1).

**Table 1:** Measurement scale for negative emotions.

Variable	Item	Measurement content
NE	A1	The content in the video makes me anxious.
	A2	The content in the video offends me.
	A3	The content in the video makes me feel like consumers are being deceived.

*Individual health threat appraisal (HTA)*: an individual’s subjective assessment of the potential harm that the food shown in the video may pose to their own and their family’s health. This scale was adapted from Bearth et al. (5) and consists of three items (Table 2).

**Table 2:** Measurement scale for individual health threat appraisal.

Variable	Item	Measurement content
HTA	B1	I feel that I or my family members might end up eating food processed in this way without even realizing it.
	B2	After watching this video, I believe that consuming food prepared in this manner poses potential health risks.
	B3	I believe that long-term consumption of the foods shown in the video increases the risk of developing certain diseases.

*Trust erosion (TE)*: the extent to which an individual’s trust in the food delivery industry, government regulation, and food labeling decreases after watching the video. This scale is adapted from Siegrist et al. (59) and Bearth et al. (5) and consists of three items after item purification (Table 3).

**Table 3:** Measurement scale for trust erosion.

Variable	Item	Measurement content
TE	C1	The video content has caused my overall trust in the food delivery industry to decline.
	C2	The video content has made me question the government regulatory agencies’ ability to ensure food safety.
	C3	Videos like these make me feel that food labels and marketing claims on the market are untrustworthy.

*Public risk cognition (PRC)*: the macro-level, stable assessment individuals form regarding the current overall food safety environment. This scale was adapted from Zhu et al. (60), with additional items developed for this study. It consists of four items (Table 4).

**Table 4:** Measurement scale for public risk cognition.

Variable	Item	Measurement content
PRC	D1	I believe that tech-fearmongering is a widespread phenomenon in the food industry.
	D2	Food safety issues are more serious than most people realize.
	D3(R)	I believe that most foods are safe and reliable (reverse-coded)
	D4	Overall, I find the current food safety situation to be concerning.

*Protective behavior intention (PBI)*: an individual’s propensity to take specific actions to avoid perceived food safety risks. This scale is based on PMT’s coping evaluation and draws upon Zhu et al. (60). It consists of five items (Table 5).

**Table 5:** Measurement scale for protective behavior intention.

Variable	Item	Measurement content
PBI	E1	I will be more mindful about choosing foods labeled no additives or all-natural.
	E2	I plan to cut back on ordering takeout or eating out.
	E3	I will buy and eat fewer pre-packaged ready-to-eat meals or takeout that seem like good value for money
	E4	I am willing to pay a higher price for food with transparent sourcing and a simple ingredient list.
	E5	I will proactively share or discuss this kind of food safety information with family and friends.

Based on the above scales, the core section of the questionnaire consists of 18 items (3 NE + 3 HTA + 3 TE + 4 PRC + 5 PBI), along with scenario validation items and demographic items, for a total of 24 items.

### 3.4 Data collection and processing

The questionnaire was distributed and collected through an online questionnaire platform, with respondents randomly assigned to one of the two conditions. After reading the assigned scenario material, respondents completed the scale items according to their immediate perceptions and feelings. The sample was recruited from Chinese adult internet users through social media and online communities, covering short-video users of different ages, educational backgrounds, and regions. Data were collected from September to November 2025. After data cleaning based on response-consistency checks, 487 valid responses were retained from 500 questionnaires, yielding an effective response rate of 97.4%. Because the sample was recruited online and is skewed toward young, highly educated women, the results should be interpreted as evidence from a platform-user survey experiment rather than as estimates for the general population.

## 4 Analysis

### 4.1 Descriptive statistics of the sample

Among the 487 valid respondents, males accounted for 23.6% and females 76.4%. The predominance of female participants is consistent with research showing that women are typically the primary decision-makers for family food procurement and tend to have higher sensitivity to food safety information. The age distribution was predominantly 19–25 years (59.2%), followed by 26–35 years (25.4%), reflecting the core user demographic of short-video platforms. Educational attainment was high, with bachelor’s degree holders comprising 68.6% and master’s degree or above comprising 24.8%. Regarding short-video viewing duration, 42.4% watched 1–2 h daily and 22.3% watched 30 min to 1 h, confirming that short videos are an integral part of participants’ daily media environment.

### 4.2 Reliability and validity tests

#### 4.2.1 Reliability test

Internal consistency reliability was assessed using Cronbach’s *α* coefficient. As shown in Table 6, all constructs exceeded the 0.70 threshold: NE (*α* = 0.951), HTA (*α* = 0.871), TE (*α* = 0.928), PRC (*α* = 0.849), and PBI (*α* = 0.788).

**Table 6:** Reliability coefficients for the measurement scales.

Dimension	Number of Items	Cronbach’s *α*
Negative Emotions (NE)	3	0.951
Individual Health Threat Appraisal (HTA)	3	0.871
Trust Erosion (TE)	3	0.928
Public Risk Cognition (PRC)	4	0.849
Protective Behavior Intention (PBI)	5	0.788

#### 4.2.2 Validity test

Exploratory factor analysis was conducted using SPSS. The KMO value was 0.864, exceeding the 0.80 threshold, and Bartlett’s test of sphericity was significant (*χ*^2^ = 12683.989, df = 231, *p <* 0.001), confirming suitability for factor analysis and demonstrating adequate structural validity (Table 7).

**Table 7:** Validity test of the scales.

Test	Statistic	Value
KMO Measure of Sampling Adequacy		0.864
Bartlett’s Test of Sphericity	Approx. Chi-Square	12683.989
	Degrees of Freedom	231
	Sig.	<0.001

#### 4.2.3 Convergent validity

Standardized factor loadings, composite reliability (CR), and average variance extracted (AVE) were computed for each construct. As shown in Table 8, all standardized factor loadings exceeded 0.60, with most above 0.80. CR values ranged from 0.858 to 0.969, all exceeding 0.70. AVE values ranged from 0.550 to 0.913, all meeting or exceeding 0.50. These results demonstrate adequate convergent validity (Table 8).

**Table 8:** Convergent validity results.

Construct	Item	Mean	SD	*λ*	CITC	*α*	CR	AVE	√AVE	k
NE	A1	3.234	1.405	0.961	0.918	0.951	0.969	0.913	0.955	3
	A2	3.314	1.573	0.942	0.880					
	A4	3.076	1.500	0.963	0.935					
HTA	B1	3.396	1.376	0.922	0.622	0.871	0.922	0.799	0.894	3
	B2	3.768	0.982	0.829	0.820					
	B3	3.487	1.373	0.928	0.782					
TE	C2	3.400	1.241	0.918	0.842	0.928	0.955	0.877	0.936	3
	C3	3.068	1.168	0.948	0.894					
	C4	3.345	1.102	0.943	0.871					
PRC	D1	4.002	1.009	0.832	0.694	0.849	0.900	0.692	0.832	4
	D2	4.105	0.940	0.845	0.717					
	D3(R)	2.766	0.798	0.744	0.552					
	D4	3.768	0.883	0.900	0.811					
PBI	E1	4.057	0.751	0.782	0.632	0.788	0.858	0.550	0.742	5
	E2	3.889	0.967	0.643	0.446					
	E3	3.959	0.961	0.817	0.701					
	E4	3.984	0.814	0.668	0.463					
	E5	3.803	0.899	0.782	0.618					

*λ* = standardized factor loading; CITC, corrected item-total correlation; D3(R) = reverse-coded; k = number of items.

### 4.3 Correlation analysis and discriminant validity

Pearson correlation analysis was conducted to examine the bivariate relationships among all study variables. As shown in Table 9, the independent variable (video type) was significantly correlated with all three mediating variables: NE (r = 0.713, *p <* 0.001), HTA (r = 0.680, *p <* 0.001), and TE (r = 0.530, *p <* 0.001). The three mediating variables were significantly and positively inter correlated (r = 0.821–0.875) and each was significantly associated with both dependent variables.

**Table 9:** Descriptive statistics and correlation analysis.

Variable	Mean	SD	1	2	3	4	5	6
1. X	–	–	–					
2. NE	3.208	1.426	0.713***	–				
3. HTA	3.550	1.121	0.680***	0.875***	–			
4. TE	3.271	1.095	0.530***	0.821***	0.861***	–		
5. PRC	3.660	0.756	0.358***	0.556***	0.707***	0.697***	–	
6. PBI	3.938	0.649	0.104*	0.311***	0.374***	0.364***	0.531***	–

X, video type (1 = tech-fearmongering, 0 = control); NE, negative emotions; HTA, health threat appraisal; TE, trust erosion; PRC, public risk cognition; PBI, protective behavior intention. ***p < 0.001, *p < 0.05.

As shown in Table 10, the square roots of AVE for each construct were all greater than the corresponding inter-construct correlations, confirming that the Fornell–Larcker criterion was fully satisfied.

**Table 10:** Fornell-Larcker criterion.

Variable	NE	HTA	TE	PRC	PBI
NE	**0.955**	0.875	0.821	0.556	0.311
HTA	0.875	**0.894**	0.861	0.707	0.374
TE	0.821	0.861	**0.936**	0.697	0.364
PRC	0.556	0.707	0.697	**0.832**	0.531
PBI	0.311	0.374	0.364	0.531	**0.742**

NE, negative emotions; HTA, health threat appraisal; TE, trust erosion; PRC, public risk cognition; PBI, protective behavior intention. Diagonal values (bold) represent? AVE. Off-diagonal values are Pearson correlation coefficients. Criterion: AVE should exceed all correlation coefficients in the corresponding row/column.

However, given the small margin of some √AVE values, this study further adopted the more stringent Heterotrait-Monotrait (HTMT) ratio criterion. The results (Table 11) indicated that HTMT ratios for two mediator pairs exceeded the 0.90 threshold: NE–HTA (0.962) and HTA–TE (0.958), while NE–TE (0.874) fell within the acceptable range. All pairs involving PRC and PBI demonstrated adequate discriminant validity (HTMT < 0.85).

**Table 11:** HTMT ratio matrix.

Variable	NE	HTA	TE	PRC	PBI
NE	–				
HTA	0.962	–			
TE	0.874	0.958	–		
PRC	0.619	0.822	0.785	–	
PBI	0.359	0.451	0.425	0.649	–

NE, negative emotions; HTA, health threat appraisal; TE, trust erosion; PRC, public risk cognition; PBI, protective behavior intention. HTMT, heterotrait-monotrait ratio. HTMT < 0.85 = strict discriminant validity; HTMT < 0.90 = lenient discriminant validity.

Several factors may account for this phenomenon. First, all variables were measured via self-report at a single time point, which may have artificially inflated inter-construct correlations due to common method variance (61). Second, from a theoretical perspective, negative emotions, health threat appraisal, and trust erosion represent highly coupled psychological processes in the context of exposure to misleading food safety information. Both the Affect Heuristic theory (37) and the risk-as-feelings hypothesis (56) posit that emotional reactions and cognitive risk judgments are inherently intertwined when individuals process threatening information, particularly in visual and emotionally charged media formats such as short videos. Third, each mediating construct was measured with only three items; short scales with high reliability tend to exhibit elevated inter-construct correlations. Despite these empirical overlaps, the Fornell–Larcker criterion was fully satisfied, and each mediator demonstrated a distinct pattern of effects on the outcome variables in the mediation analysis (see Section 4.5), providing evidence of nomological validity supporting their theoretical distinction. Supplementary robustness checks, including VIF diagnostics, ridge regression, and a higher-order composite mediator model, are reported in the Supplementary material.

### 4.4 Regression analysis

Linear regression analysis confirmed that tech-fearmongering food production short videos significantly and positively predicted all three mediators, as shown in Table 12. Hypotheses H1, H2, and H3 are thus supported.

**Table 12:** Regression of video type on mediating variables.

Path	B	SE	*β*	*t*	*p*	R^2^	F
X → NE	2.041	0.091	0.713	22.432	<0.001	0.509	503.19
X → HTA	1.530	0.075	0.680	20.452	<0.001	0.463	418.27
X → TE	1.155	0.085	0.530	13.611	<0.001	0.276	185.27

X, video type (1 = tech-fearmongering, 0 = control); NE, negative emotions; HTA, health threat appraisal; TE, trust erosion; PRC, public risk cognition; PBI, protective behavior intention. All VIF < 10.

### 4.5 Mediation effect test

This study employed the PROCESS macro (Hayes, 2018) Model 4 with 5,000 bootstrap resamples to test the mediation effects. Given the moderately high inter-correlations among the three mediators, both single-mediator models (testing each pathway independently, capturing total mediating capacity) and a parallel multiple mediator model (entering all three simultaneously, testing unique contributions) are reported.

#### 4.5.1 Single-mediator models

As shown in Table 13, all six single-mediator pathways exhibited significant positive indirect effects with bootstrap confidence intervals excluding zero. For PRC, HTA showed the largest indirect effect (0.890), followed by NE (0.662) and TE (0.561). For PBI, HTA showed the strongest effect (0.500), followed by NE (0.448) and TE (0.292). All pathways exhibited a suppression pattern: the indirect effects were positive while the direct effects were negative. This pattern suggests that the entertainment-oriented presentation of such videos may directly weaken risk cognition and protective intentions, while the cognitive, emotional, and trust-related mechanisms can counteract this direct effect and drive heightened risk awareness and protective action. The result should be interpreted as a mechanism observed under scenario-based exposure, not as evidence of long-term behavioral change.

**Table 13:** Single-mediator mediation results.

Path	Total effect	Direct effect	Indirect effect	BootSE	BootLLCI	BootULCI	Type
DV = public risk cognition (PRC)							
X → NE → PRC	0.543	−0.120	0.662	0.066	0.537	0.798	Sup.
X → HTA → PRC	0.543	−0.347	0.890	0.053	0.785	0.995	Sup.
X → TE → PRC	0.543	−0.018	0.561	0.054	0.456	0.668	Sup.
DV = protective behavior intention (PBI)							
X → NE → PBI	0.135	−0.313	0.448	0.051	0.346	0.549	Sup.
X → HTA → PBI	0.135	−0.365	0.500	0.045	0.415	0.589	Sup.
X → TE → PBI	0.135	−0.157	0.292	0.039	0.217	0.370	Sup.

X, video type (1 = tech-fearmongering, 0 = control); NE, negative emotions; HTA, health threat appraisal; TE, trust erosion; PRC, public risk cognition; PBI, protective behavior intention. Sup., suppression effect (indirect and direct effects have opposite signs). All indirect effect CIs exclude zero. Bootstrap = 5,000 resamples.

#### 4.5.2 Parallel multiple mediator model

To test the unique contribution of each mediator while controlling for the others, a parallel model was estimated. Given the high inter-correlations, this model provides a more conservative estimate. The effects in the parallel model should therefore be interpreted as the unique contribution of each mediator after shared variance with the other mediators has been controlled, rather than as a replacement for the total mediating capacity shown in the single-mediator models.

In the parallel model for PRC (Table 14), all three unique indirect pathways were statistically significant. HTA contributed the largest unique indirect effect (0.747), followed by TE (0.320). The NE pathway showed a negative sign (−0.353), which is a well-documented statistical suppression phenomenon that occurs when highly correlated mediators compete for shared variance in a simultaneous model (71). The single-mediator model (Table 13) confirms the NE pathway is positive (0.662). For PBI (Table 15), only the HTA pathway was significant (0.400), while NE and TE did not reach significance in the parallel model due to shared variance absorption. These results indicate the unique effects remaining after controlling for the other mediators and should not be read as evidence that NE or TE lacks total mediating capacity.

**Table 14:** Parallel mediation results: DV = PRC.

Path	Effect	BootSE	*t*	BootLLCI	BootULCI	Result
Total Effect (c)	0.543	0.064	8.441	0.416	0.669	Sig.
Direct Effect (c′)	−0.170	0.068	−2.494	−0.305	−0.036	Sig.
Total Indirect	0.713	0.055	–	0.604	0.821	Sig.
X → NE → PRC	−0.353	0.065	–	−0.480	−0.222	Sig.†
X → HTA → PRC	0.747	0.066	–	0.609	0.866	Sig.
X → TE → PRC	0.320	0.055	–	0.216	0.433	Sig.

X, video type (1 = tech-fearmongering, 0 = control); NE, negative emotions; HTA, health threat appraisal; TE, trust erosion; PRC, public risk cognition. Bootstrap = 5,000 resamples.

**Table 15:** Parallel mediation results: DV = PBI.

Path	Effect	BootSE	*t*	BootLLCI	BootULCI	Result
Total Effect (c)	0.135	0.059	2.298	0.020	0.251	Sig.
Direct Effect (c′)	−0.366	0.081	−4.541	−0.525	−0.208	Sig.
Total Indirect	0.502	0.049	–	0.407	0.600	Sig.
X → NE → PBI	0.044	0.083	–	−0.120	0.212	NS
X → HTA → PBI	0.400	0.087	–	0.223	0.564	Sig.
X → TE → PBI	0.057	0.061	–	−0.061	0.178	NS

X, video type (1 = tech-fearmongering, 0 = control); NE, negative emotions; HTA, health threat appraisal; TE, trust erosion; PBI, protective behavior intention. The negative NE indirect effect in the parallel model reflects a statistical suppression artifact due to shared variance among the three correlated mediators. NS, not significant. Bootstrap = 5,000 resamples.

Table 16 summarizes the hypothesis testing results.

**Table 16:** Summary of hypothesis testing results.

Hypothesis	Content	Evidence	Supported?
H1	Videos → HTA (+)	B = 1.530, *p <* 0.001	Yes
H1a	Videos → HTA → PRC (+)	Parallel: 0.747 [0.609, 0.866]	Yes
H1b	Videos → HTA → PBI (+)	Parallel: 0.400 [0.223, 0.564]	Yes
H2	Videos → NE (+)	B = 2.041, *p <* 0.001	Yes
H2a	Videos → NE → PRC (+)	Single: 0.662 [0.537, 0.798]	Yes
H2b	Videos → NE → PBI (+)	Single: 0.448 [0.346, 0.549]	Yes
H3	Videos → TE (+)	B = 1.155, *p <* 0.001	Yes
H3a	Videos → TE → PRC (+)	Parallel: 0.320 [0.216, 0.433]	Yes
H3b	Videos → TE → PBI (+)	Single: 0.292 [0.217, 0.370]	Yes

NE, negative emotions; HTA, health threat appraisal; TE, trust erosion; PRC, public risk cognition; PBI, protective behavior intention. For pathways where the parallel model produced suppression artifacts (H2a, H2b, H3b), single-mediator results are cited. For pathways supported in both models (H1a, H1b, H3a), the parallel model (more conservative) is cited.

## 5 Results and discussion

### 5.1 Research summary

Focusing on tech-fearmongering food production short videos as a case of misleading health risk information on digital platforms, this study collected 487 valid responses through a scenario-based experiment. Cronbach’s alpha coefficients, exploratory factor analysis, and convergent validity indicators, including CR and AVE, met conventional thresholds, indicating acceptable measurement quality. The Fornell-Larcker criterion supported discriminant validity, while the supplementary HTMT analysis also showed that the three mediators were highly correlated. This overlap is important for interpretation because negative emotions, health threat appraisal, and trust erosion may operate as closely connected psychological responses when users encounter visually salient health risk information.

The most important finding is the recurring suppression pattern across the single-mediator models. This pattern reveals an internal tension in tech-fearmongering food production short videos. As a form of misleading health risk information, these videos rely on visual shock and emotional resonance, but their entertainment-oriented format may also reduce the perceived seriousness of the risk message.

This contradiction is related to the interaction between topic seriousness and entertainment format. On the one hand, sensational or joking presentation may directly weaken risk perception and protective behavioral intention. On the other hand, the same content can indirectly strengthen public health decision-making by triggering health threat appraisal, negative emotions, and trust erosion. Taken together, this finding provides an integrated framework for understanding how misleading risk information operates in digital public health communication.

This finding provides an integrated analytical framework for understanding the communication mechanism of tech-fearmongering food production short videos and extends the explanatory boundary of PMT in the context of misleading health risk information on digital platforms.

#### 5.1.1 Health threat appraisal as the core of protective motivation

HTA turned out to be the strongest unique mediator in the parallel model, yielding the largest indirect effects on both PRC (0.747) and PBI (0.400). This result lends direct support to PMT’s central claim that threat assessment sits at the core of protective motivation. At the same time, it broadens the scope of this claim by showing that it holds in a digital media setting where the threat information in question is misleading food safety content rather than conventional health warnings.

#### 5.1.2 Negative emotions as an independent explanatory pathway

The emotional pathway also carries independent explanatory weight. In the single-mediator models, NE produced sizable indirect effects on PRC (0.662) and PBI (0.448). This shows that when misinformation about food safety is disseminated through media that rely heavily on sensory impact, such as short videos, affective responses can rival cognitive appraisal in shaping audience reactions. This observation is consistent with the Affect Heuristic framework and the risk-as-feelings hypothesis, and it suggests that PMT should more explicitly incorporate an affective channel alongside its traditional cognitive emphasis.

#### 5.1.3 Trust erosion as a social trust pathway

TE yielded a significant unique indirect effect on PRC in the parallel model (0.320) and also demonstrated significant total mediating capacity for both dependent variables in the single-mediator analyses. This suggests that response evaluation in PMT should not be treated as a purely individual-level cognitive process. It also depends on a broader social trust foundation. When trust in the food industry, platform information, or regulatory systems is weakened, the public may have more difficulty evaluating which protective responses are effective.

#### 5.1.4 Media format as a boundary condition

Their abstract, sensational, and entertainment-oriented imagery may weaken the seriousness of health risk information. The observed net effect depends on whether cognitive, emotional, and trust-related mechanisms are strong enough to overcome the noise introduced by the media format.

### 5.2 Practical implications

Based on the findings, countermeasures should focus on digital public health risk communication and platform content governance. Tech-fearmongering food production short videos can shape public decision-making through risk perception, negative emotions, and trust erosion, while their entertainment-oriented format may weaken risk understanding. Effective governance should therefore combine transparent information disclosure, authoritative risk communication, algorithmic content governance, and early warning mechanisms for misleading health risk information.

#### 5.2.1 Promoting transparency in the food industry

Promoting a shift in the food industry from passive defense to active communication is important for repairing system trust. Trust reconstruction depends on supply chain transparency, risk communication optimization, and proactive trust repair.

At the level of supply chain transparency, food enterprises can establish public open days or use online live broadcasting and short videos to present back-end production processes (69). Visual evidence can help reduce the information gap around food production, improve public understanding, and weaken misleading narratives that grow out of uncertainty and opacity (62, 63).

At the narrative and communication level, risk communication should move from an antagonistic narrative to a co-construction narrative. Compared with simple rebuttal, alternative narrative frameworks can reduce cognitive load and make corrective information easier to process. Food enterprises and public health communicators can produce high-quality short videos explaining food additives and production processes, thereby increasing prior knowledge and improving users’ ability to identify misleading claims (64).

The concept of trust repair should be embedded in communication practices. The findings show that tech-fearmongering food production short videos affect brand trust and system trust, thereby shaping coping appraisal. Food enterprises and regulators should move beyond simple fact release and demonstrate the reliability of safety assurance mechanisms, including standard setting, transparent regulatory implementation, and penalties for violations (49, 65). Shifting communication from static safety claims to process credibility can send clearer response-efficacy signals and strengthen trust in the broader risk response system.

#### 5.2.2 Strengthening self-media regulation and platform governance

At the content creator level, self-media bloggers should recognize their social responsibilities as information disseminators. In the short-video field, traffic-oriented production can encourage sensational presentation, yet source professionalism and credibility remain key factors shaping public risk perception and information adoption (66). Food-related creators should therefore prioritize content quality, follow communication ethics, verify information before publication, and avoid inflammatory visual elements or absolute narratives that may cause unnecessary panic (67).

At the level of platform governance, short-video platforms should move beyond general content review and establish specific governance mechanisms for health and safety risk information. Traffic-oriented recommendation logic may increase user engagement in the short term, but it can also amplify misleading content. Platforms should optimize recommendation algorithms, limit the circulation of identified high-risk misleading content, increase the visibility of evidence-based authoritative content, and cooperate with professional institutions to establish rapid verification channels for food safety information (9, 63).

At the level of collaborative governance, platforms should establish cooperative correction mechanisms with professional institutions (68). A warning and case database for misleading health risk information can help move governance from *post-hoc* refutation to pre-warning (70). Platforms can also provide food safety communication resources for creators, improving the content ecology at the source.

### 5.3 Limitations and future directions

Several limitations should be acknowledged. First, the data came from a cross-sectional scenario-based experiment. This design helps identify short-term responses under controlled exposure, but it cannot show how risk perception, emotion, trust, and behavior evolve over time. Second, the stimulus was a written scenario adapted from short-video content rather than direct exposure to real videos; future studies should use real platform videos or field experiments to improve ecological validity. Third, the sample was skewed toward young, highly educated women. This profile partly matches active short-video users and food-related decision-makers, but it constrains generalizability. Fourth, the three mediators were highly intercorrelated, with r = 0.821–0.875 and HTMT = 0.874–0.962. This overlap is theoretically understandable because cognition, emotion, and trust are intertwined when users encounter threatening health risk information, but it still creates discriminant validity and shared-variance concerns. Fifth, the analysis relies on self-reported outcome variables, so common method bias and social desirability bias cannot be fully excluded. Future research should combine longitudinal designs, behavioral platform data, real video stimuli, and group comparisons by digital literacy and food science knowledge.

## 6 Conclusion

Digital platforms have become important sites of exposure to health risk information, making misleading food-related content a digital public health governance issue. This study applies PMT to tech-fearmongering food production short videos and shows how cognitive appraisal, negative emotions, and trust erosion jointly shape public risk cognition and protective behavioral intention. The findings extend PMT by showing that affective and trust-based channels matter in digital media environments, while also supporting platform governance, transparent risk communication, and health information quality control as practical priorities.

## Data Availability

The raw data supporting the conclusions of this article will be made available by the authors, without undue reservation.
