# Supplementary material for: Misleading health risk information and public health decision-making on digital platforms: evidence from tech-fearmongering food production short videos
Source: Front Public Health. 2026 Jul 20;14:1894652. doi: 10.3389/fpubh.2026.1894652 (PMC13429709; doi:10.3389/fpubh.2026.1894652)
Supplement: Supplementary file 1 [file Table_1.docx]

Supplementary Material

# Supplementary Tables

This supplementary document addresses potential multicollinearity concerns among the three mediators (NE, HTA, TE) identified during the review process. We report VIF diagnostics, ridge regression robustness checks, bootstrap confidence intervals, sensitivity analyses, and a higher-order composite mediator model.

## S1. Variance Inflation Factor (VIF) Diagnostics

To quantify the severity of multicollinearity in the parallel mediation model, VIF values were computed for both outcome equations. Results are presented in Table S1.

**Table S1 VIF Values in the Parallel Mediation Models**

| Variable | VIF (DV = PRC) | VIF (DV = PBI) |
| --- | --- | --- |
| Video Type (X) | 2.27 | 2.30 |
| NE | 5.49 | 5.73 |
| HTA | 6.34 | 7.55 |
| TE | 4.53 | 4.91 |
| PRC | — | 2.32 |
| Threshold | < 10 | < 10 |

*Note: VIF < 5 indicates low collinearity; VIF 5-10 indicates moderate collinearity; VIF > 10 indicates severe collinearity (Hair et al., 2019). All VIF values fall below the critical threshold of 10, though NE (5.49/5.73) and HTA (6.34/7.55) indicate moderate collinearity consistent with the high inter-mediator correlations reported in Table 4-5.*

## S2. Ridge Regression Robustness Check

To verify that OLS coefficient estimates are not unduly distorted by multicollinearity, ridge regression was employed as a robustness check. Ridge regression introduces a small bias (via the penalty parameter alpha) to stabilize coefficient estimates when predictors are highly correlated. The optimal alpha was selected via 5-fold cross-validation.

**Table S2 OLS vs. Ridge Regression Comparison (DV = PRC, Standardized Coefficients)**

| Variable | OLS beta (std.) | Ridge beta (std.) | Direction Consistent |
| --- | --- | --- | --- |
| Video Type (X) | -0.0848 | -0.0847 | Yes |
| NE | -0.2467 | -0.2442 | Yes |
| HTA | 0.5463 | 0.5437 | Yes |
| TE | 0.3031 | 0.3030 | Yes |
| Optimal alpha | — | 0.4037 |  |

*Note: Optimal ridge penalty alpha = 0.4037 selected via 5-fold cross-validation. All coefficient signs and relative magnitudes are consistent between OLS and ridge estimates, indicating that multicollinearity does not distort the substantive conclusions. OLS R2 = 0.5692; Ridge R2 = 0.5692.*

## S3. Bootstrap Confidence Intervals for Indirect Effects

Bias-corrected bootstrap confidence intervals (5,000 resamples) were computed for each single-mediator indirect effect to provide nonparametric inference that does not assume normality of the indirect effect distribution.

**Table S3 Bootstrap Indirect Effects (Single-Mediator Models, 5,000 Resamples)**

| Indirect Path | Effect | Boot SE | Boot ULCI | Significant |
| --- | --- | --- | --- | --- |
| X -> NE -> PRC | 0.661 | 0.066 | 0.798 | Yes |
| X -> NE -> PBI | 0.447 | 0.052 | 0.553 | Yes |
| X -> HTA -> PRC | 0.886 | 0.054 | 0.991 | Yes |
| X -> HTA -> PBI | 0.500 | 0.045 | 0.592 | Yes |
| X -> TE -> PRC | 0.557 | 0.053 | 0.665 | Yes |
| X -> TE -> PBI | 0.292 | 0.039 | 0.370 | Yes |

*Note: Bootstrap resamples = 5,000. 95% bias-corrected confidence intervals. An indirect effect is significant if the CI excludes zero. All six single-mediator indirect effects are significant, confirming the mediation pathways reported in Table 4-8.*

## S4. Sensitivity Analysis: Reduced Mediator Models

To assess whether the substantive conclusions are robust to model specification, three two-mediator models were estimated, each omitting one mediator. This sensitivity analysis tests whether the parallel model findings are driven by collinearity artifacts.

**Table S4 Sensitivity Analysis - Two-Mediator Models (DV = PRC)**

| Model | Variable | beta | SE | t | p | R2 |
| --- | --- | --- | --- | --- | --- | --- |
| NE + HTA (TE omitted) | Video Type (X) | -0.290 | 0.068 | -4.260 | 0.0000*** | 0.5337 |
|  | NE | -0.091 | 0.036 | -2.508 | 0.0125* |  |
|  | HTA | 0.665 | 0.044 | 15.153 | 0.0000*** |  |
| NE + TE (HTA omitted) | Video Type (X) | 0.018 | 0.071 | 0.251 | 0.8020 | 0.4867 |
|  | NE | -0.032 | 0.037 | -0.864 | 0.3882 |  |
|  | TE | 0.511 | 0.040 | 12.819 | 0.0000*** |  |
| HTA + TE (NE omitted) | Video Type (X) | -0.297 | 0.064 | -4.640 | 0.0000*** | 0.5498 |
|  | HTA | 0.394 | 0.048 | 8.282 | 0.0000*** |  |
|  | TE | 0.205 | 0.042 | 4.877 | 0.0000*** |  |

*Note: *** p < .001, ** p < .01, * p < .05. HTA and TE remain significant positive predictors across all specifications. NE shows a negative coefficient, consistent with the suppression effect observed in the full parallel model. The pattern of results is stable across model specifications.*

## S5. Higher-Order Composite Mediator Model

Given the high inter-correlations among mediators, a composite "Negative Appraisal" variable was created by averaging NE, HTA, and TE scores. This higher-order construct captures the shared variance among the three mediators while eliminating collinearity. Principal Component Analysis confirmed that a single component explains 90.2% of the variance in the three mediators (loadings: NE = 0.575, HTA = 0.584, TE = 0.572), supporting the unidimensional composite.

**Table S5 Higher-Order Composite Mediator Model Results**

| Path | Effect | SE | t / Boot SE | p / 95% CI | Result |
| --- | --- | --- | --- | --- | --- |
| a: X -> Composite | 1.575 | 0.077 | 20.488 | 0.0000*** | Sig. |
| b: Composite -> PRC | 0.532 | 0.029 | 18.149 | 0.0000*** | Sig. |
| b: Composite -> PBI | 0.308 | 0.032 | 9.691 | 0.0000*** | Sig. |
| c': X -> PRC (direct) | -0.296 | 0.068 | -4.359 | 0.0000*** | Sig. |
| c': X -> PBI (direct) | -0.351 | 0.074 | -4.764 | 0.0000*** | Sig. |
| Indirect: X -> Comp -> PRC | 0.836 |  | 0.068 | [0.708, 0.979] | Sig. |
| Indirect: X -> Comp -> PBI | 0.485 |  | 0.049 | [0.393, 0.586] | Sig. |

*Note: Composite = mean(NE, HTA, TE). Bootstrap resamples = 5,000 for indirect effects. Both indirect pathways via the composite mediator are significant (CIs exclude zero), confirming that the collective "negative appraisal" mechanism mediates the effect of tech-fearmongering videos on both public risk cognition and protective behavioral intention.*

## S6. Reliability of Modified Scales (Post-Item Deletion)

Following the deletion of items A3, A5, and C1 based on cross-loading criteria, the internal consistency of all modified scales was re-assessed using Cronbach's alpha.

**Table S6 Reliability Coefficients for Modified Scales**

| Scale | Items | k | Cronbach's alpha |
| --- | --- | --- | --- |
| NE (Negative Emotions) | A1, A2, A4 | 3 | 0.951 |
| HTA (Health Threat Appraisal) | B1, B2, B3 | 3 | 0.871 |
| TE (Trust Erosion) | C2, C3, C4 | 3 | 0.928 |
| PRC (Public Risk Cognition) | D1, D2, D3(R), D4 | 4 | 0.849 |
| PBI (Protective Behavioral Intention) | E1, E2, E3, E4, E5 | 5 | 0.788 |

*Note: All Cronbach's alpha values exceed the 0.70 threshold (Nunnally & Bernstein, 1994), confirming adequate internal consistency for the modified scales.*

## S7. Discussion of Multicollinearity and Discriminant Validity

The high inter-correlations among NE, HTA, and TE (r = 0.821-0.875) and corresponding HTMT values (0.911-0.962) warrant careful interpretation. Several methodological and theoretical considerations are relevant:

1. Theoretical justification. Negative emotions (NE), health threat appraisal (HTA), and trust erosion (TE) are theoretically distinct but empirically coupled constructs in the context of risk communication. Appraisal theories of emotion (Lazarus, 1991; Lerner & Keltner, 2000) predict that emotional responses and cognitive risk assessments co-occur and mutually reinforce each other when individuals encounter threatening health information. The strong correlations reflect genuine psychological coupling rather than measurement redundancy.

2. Common method variance. All mediators were measured via self-report Likert scales in the same survey session, which may inflate shared variance.

3. Robustness of findings. The following robustness checks converge on the same substantive conclusion - that tech-fearmongering videos increase public risk cognition and protective behavioral intentions through negative appraisal mechanisms:

(a) VIF values (5.49-7.55) remain below the critical threshold of 10 (Table S1).

(b) Ridge regression reproduces identical coefficient signs and comparable magnitudes (Table S2).

(c) All six single-mediator bootstrap CIs exclude zero (Table S3).

(d) Sensitivity analyses with reduced mediator sets yield consistent patterns (Table S4).

(e) A higher-order composite mediator model confirms significant mediation without collinearity (Table S5).

4. Recommendation for interpretation. Given the above, we recommend that the parallel mediation results be interpreted with the caveat that unique mediator contributions (especially the suppression effect on NE to PRC) should be treated as tentative. The single-mediator models provide the primary evidence for each individual pathway, while the parallel model serves as a complementary test of relative contributions.
